# The Role and Mechanism of Histone Deacetylases in Acute Kidney Injury

**DOI:** 10.3389/fphar.2021.695237

**Published:** 2021-06-16

**Authors:** Xun Zhou, Hui Chen, Yingfeng Shi, Xiaoyan Ma, Shougang Zhuang, Na Liu

**Affiliations:** ^1^ Department of Nephrology, Shanghai East Hospital, Tongji University School of Medicine, Shanghai, China; ^2^ Department of Medicine, Rhode Island Hospital and Alpert Medical School, Brown University, Providence, RI, United States

**Keywords:** acute kidney injury, histone deacetylase, epigenetic modification, mitochondrial biogenesis, oxidative stress

## Abstract

Acute kidney injury (AKI) is a common clinical complication with an incidence of up to 8–18% in hospitalized patients. AKI is also a complication of COVID-19 patients and is associated with an increased risk of death. In recent years, numerous studies have suggested that epigenetic regulation is critically involved in the pathophysiological process and prognosis of AKI. Histone acetylation, one of the epigenetic regulations, is negatively regulated by histone deacetylases (HDACs). Increasing evidence indicates that HDACs play an important role in the pathophysiological development of AKI by regulation of apoptosis, inflammation, oxidative stress, fibrosis, cell survival, autophagy, ATP production, and mitochondrial biogenesis (MB). In this review, we summarize and discuss the role and mechanism of HDACs in the pathogenesis of AKI.

## Introduction

AKI is a rapid loss of renal function characterized by inflammation and cell death, ultimately leading to changes in renal function and structure (Mehta et al., 2007; Wen et al., 2010). According to Grampian Laboratory Outcomes Morbidity and Mortality Study II (Sawhney et al., 2017), AKI incidence in hospitalized patients is reported to be between 8 and 18% and associated with increased mortality. In a multicenter survey of AKI in China in 2013, an estimated 1.4–2.9 million AKI patients were hospitalized, with a hospital mortality rate of 12.4% (Yang et al., 2015). Even after complete recovery of renal function, the survival rates of AKI patients were lower than the survival rates of non-AKI patients (Xu et al., 2015). In a meta-analysis (Hansrivijit et al., 2020), the incidence of AKI in overall patients with COVID-19 is 8.3%, and AKI is also associated with an increased risk of death in critically ill patients with COVID-19.

There have been many studies on the mechanisms of AKI. Renal ischemia and subsequent reperfusion injury induce signaling cascades that lead to renal cell necrosis, apoptosis, and inflammation, ultimately leading to AKI (Malek and Nematbakhsh, 2015; Han and Lee, 2019). Inflammatory cascades are caused by endothelial cell injury, activation, and interaction with white blood cells through adhesion molecules (Sheridan and Bonventre, 2000). Renal ischemia reperfusion injury (IRI) can lead to the destruction of perivascular matrix, thus leading to increased permeability of endothelial barrier (Molitoris and Sutton, 2004). IRI can also up-regulate a variety of adhesion molecules, and the activated white blood cells can bind to endothelial cells through adhesion molecules and further damage endothelial cells (Kelly and Molitoris, 2000). Renal tubular epithelial cells also regulate inflammation by producing a variety of pro-inflammatory cytokines and chemokines (Sharfuddin and Molitoris, 2011). After renal ischemia, neutrophils accumulate in the kidney, secreting pro-inflammatory cytokines and chemokines, releasing reactive oxidative species (ROS) after reperfusion. Oxidative stress caused by ROS can change the oxidative phosphorylation of mitochondria; mitochondrial dysfunction leads to reduced adenosine triphosphate (ATP) synthesis (Malis and Bonventre, 1988; Bonventre, 1993; Johnson and Weinberg, 1993; Paller, 1994). In recent years, a large number of literature studies have reported that AKI is regulated by epigenetics (Tang and Zhuang, 2015; Tang and Zhuang, 2019).

Epigenetics refers to heritable changes in the function of a gene without changes in its DNA sequence (including acetylation, methylation, phosphorylation, ubiquitination, carbonylation, glycosylation, and microRNA expression). Histone deacetylases (HDACs) can remove acetyl groups from the ε-amino groups of lysine residues of histones and non-histones, causing condensation of chromatin structures (Roth et al., 2001; Thiagalingam et al., 2003). HDACs are classified into four classes: class I HDACs (HDAC1, 2, 3, and 8); class II HDACs that are subdivided into two groups, IIA (HDAC4, 5, 7, 9) and IIB (HDAC6 and 10); class III HDACs that are also called sirtuins (SIRT1–SIRT7); and class IV HDAC (HDAC11) (Tang and Zhuang, 2015; Tang and Zhuang, 2019). HDACs are essential for maintaining a dynamic equilibrium of protein acetylation. Histone acetylation is commonly associated with the transcriptional activation of genes and plays a critical role in the formation of local “open chromatin” structures required for binding to multiple transcription factors (Sterner and Berger, 2000). Lysine acetylation can open chromatin structures and enhance transcription activation (Autin et al., 2019). In contrast, the removal of acetyl groups by HDACs frequently suppresses the activity of genes (Seto and Yoshida, 2014). However, non-histone protein lysine acetylation plays a diverse role in the regulation of all aspects of cellular processes that may result in transcription activation or repression (Yang and Seto, 2007; Smith, 2008; Jian et al., 2017). Previous studies have shown that disruption of HDAC activity can lead to uncontrolled proliferation, inflammation, and fibrosis (Hyndman, 2020).

From RNA-Seq (Chen et al., 2017; Lee et al., 2015) and microarray (Yu et al., 2009) studies, all of the HDACs are expressed in the nephron. Hyndman and Knepper mapped the expressions of histone acetyltransferases (HATs) and HDACs along the nephron based on rat transcriptomic data; the collecting duct (both cortical and inner medullary) had high expression of the lysine deacetylases (HDAC1–5, HDAC10, HDAC11, SIRT2–7) (Hyndman and Knepper, 2017). Numerous studies have examined AKI-induced histone acetylation changes in the kidney (Table 1). Hyndman et al. (2019) first performed immunohistochemical analyses determining the localization of the class I, II, and IV HDACs in the kidney cortex of male bilateral IRI mice, and the most significant changes observed were with HDAC1 (class I) and HDAC4 (class II). This review article will summarize the current studies on the role and mechanism of HDACs in AKI.

**TABLE 1:** HDAC alterations in AKI.

HDAC class	Renal injury	Compartment	Assay(s) use	Chromatin response to AKI	Ref.
I	Bilateral I/R (severe)	Male C57BL/6 mouse kidney	Western blot	↑HDAC1	Hyndman et al. (2019)
	Cisplatin	Male C57BL/6 mouse kidney	Real-time PCR	↑HDAC1, HDAC2	Ranganathan et al. (2016)
	UUO	Male C57BL/6 mouse kidney	Western blot, immunohistochemistry	↑HDAC1, HDAC2	Marumo et al. (2010)
	Endotoxin	Male BALB/c mouse kidney	Western blot	↑HDAC2	Hsing et al. (2012)
II	Unilateral I/R (severe), ATP depletion	Female C57BL6 mouse kidney	Western blot, immunohistochemistry	↓HDAC5	Marumo et al. (2008)
	Bilateral I/R (severe)	Male C57BL/6 mouse kidney	Western blot	↑HDAC4	Hyndman et al. (2019)
	Endotoxin	Male BALB/c mouse kidney	Western blot	↑HDAC5	Hsing et al. (2012)
	Cisplatin	Male C57BL/6 mouse kidney	Real-time PCR	↑HDAC6	Ranganathan et al. (2016)
	UUO	Male C57BL/6 mouse kidney	Western blot	↑HDAC4,HDAC5, HDAC6, HDAC10	Choi et al. (2016)
III	Bilateral I/R (severe)	Male C57BL/6 mouse kidney	Western blot, real-time PCR	↑SIRT1	Fan et al. (2013)
	Bilateral I/R (mild)	Male Sprague Dawley rat kidney	Western blot	↑SIRT1	Funk and Schnellmann (2013)
	Bilateral I/R (severe)	Male Wistar rat kidney	Western blot	↓SIRT3	Peerapanyasut et al. (2020)
	Unilateral I/R (severe)	Male C57BL/6 mouse kidney	Immunohistochemistry	↓SIRT6	Gao et al. (2020)
	Cisplatin	Female C57BL6 mouse kidney	Real-time PCR	↓SIRT3	Morigi et al. (2015)
		Male C57BL/6 mouse kidney	Real-time PCR	↑SIRT7	Miyasato et al. (2018)
		Male C57BL/6 mouse kidney	Real-time PCR	↑SIRT3, SIRT6	Ranganathan et al. (2016)
				↓SIRT4, SIRT5	
	Sepsis-induced	Male C57BL/6 mouse kidney	Western blot	↑SIRT1	Deng et al. (2021)
		Male C57BL/6 mouse kidney	Western blot	↓SIRT3	Zhao et al. (2016)
IV	Unilateral I/R (severe)	Male C57BL/6 mouse kidney	Western blot	↓HDAC11	Kim et al. (2013)

## Class I Histone Deacetylases and Acute Kidney Injury

Class I HDACs are essential for gene expression, growth, and differentiation in the kidney and are closely related to the regulation of many key developmental pathways (Chen et al., 2011). Our previous study (Tang et al., 2013) found that the inhibition of class I HDAC activity by the selective inhibitor MS-275 decreased the proliferation and cyclin expression of cultured renal proximal tubular cells (RPTCs). RPTC proliferation requires activation of the epidermal growth factor receptor (EGFR) as well as signal transduction and transcriptional activator 3 (STAT3). Inhibition of class I HDACs results in reduced phosphorylation and expression of the EGFR and reduced phosphorylation of STAT3. In addition, STAT3 functions downstream of the EGFR, and EGFR blocking further inhibits STAT3 phosphorylation. This study suggests that class I HDACs regulate renal epithelial cell proliferation through activation of the EGFR/STAT3 signaling pathway. In folic acid and rhabdomyolysis models of AKI, we found that inhibiting class I HDACs with MS-275 (Tang et al., 2014) leads to more severe renal tubular damage. Blocking the activity of class I HDACs impairs renal regeneration. Renal injury is associated with increased EGFR, STAT3, and Akt phosphorylation. Treatment with MS-275 inhibits EGFR, STAT3, and Akt phosphorylation. Therefore, activation of class I HDACs is involved in renal protection and functional recovery by regulating the EGFR/STAT3 signaling pathway and Akt phosphorylation, essential for renal regeneration after AKI.

In contrast to the aforementioned results, MS-275 reduced kidney damage and improved the survival rate of mice in a model of lipopolysaccharide (LPS)-induced AKI through inhibiting ROS stress and endoplasmic reticulum (ER) stress (Zhang et al., 2018). In another study, the same results were obtained. Treatment with romidepsin (FK228, a selective inhibitor of HDAC1 and HDAC2) significantly reduced the kidney injury induced by LPS, *via* downregulating the expression of CYP2E1 by inhibiting the binding of hepatocyte nuclear factor-1α with the CYP2E1 promoter (Cheng et al., 2020). In cisplatin-induced AKI models, class I–selective HDAC inhibitors (HDACi) can improve kidney function. Treatment with MS-275 resulted in a significant increase in activated microglia/macrophage WAP domain protein (AMWAP) expression in proximal tubular cells, further reducing inflammation and apoptosis (Ranganathan et al., 2016). In cisplatin-induced AKI, overexpression of HDAC2 promotes cisplatin-treated tubular epithelium cell apoptosis, and inhibition of HDAC2 promotes the level of bone morphogenetic protein-7 (BMP-7), thus suppressing epithelial cell apoptosis (Zhang et al., 2020).

**FIGURE 1:**
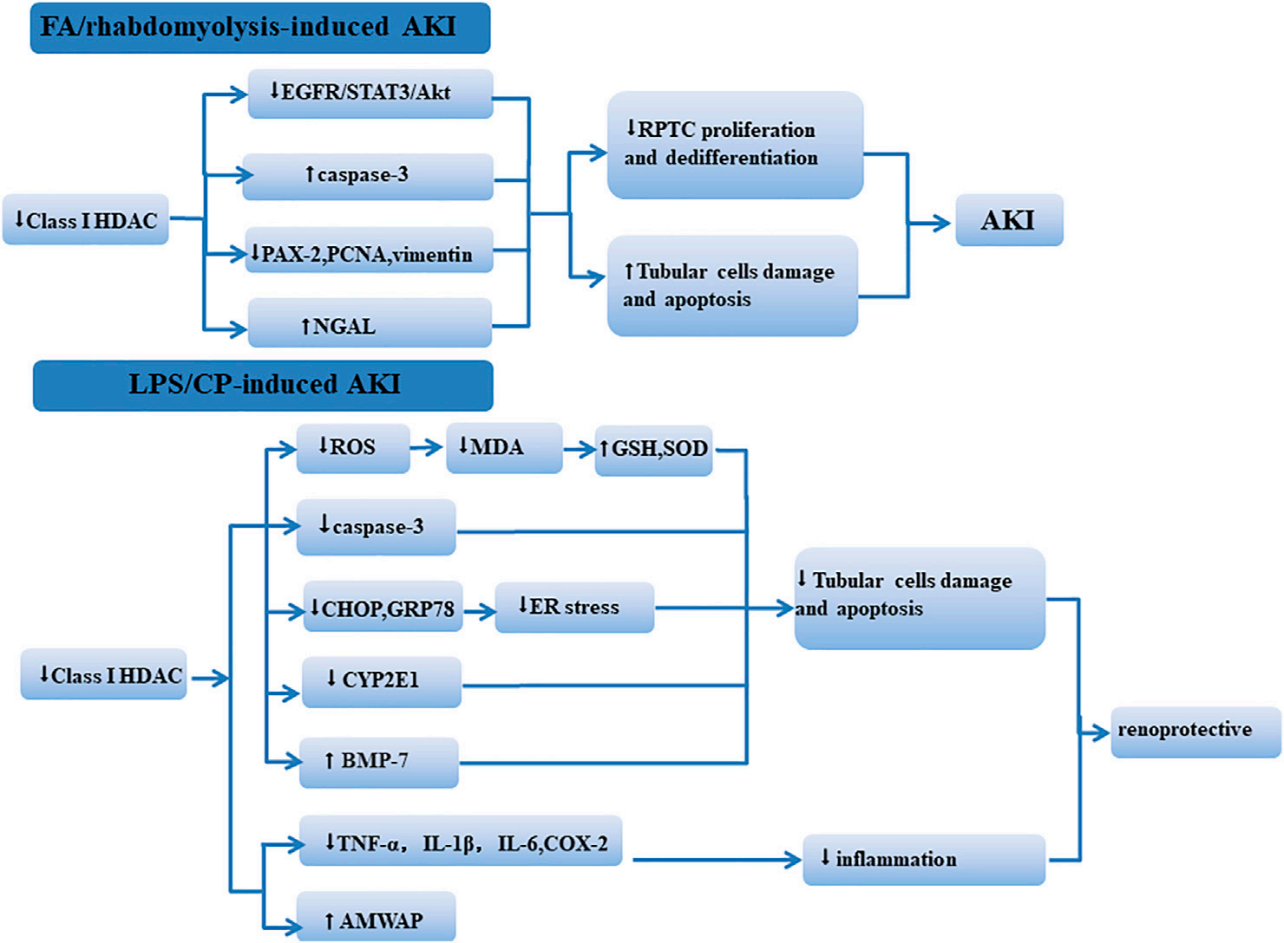
Mechanism of class I HDACs influencing the kidney in different AKI models. In FA/rhabdomyolysis-induced AKI, class I HDACs participate in renal protection by regulating the EGFR/STAT3/AKt signaling pathway and reducing tubular cell apoptosis. In LPS/CP-induced AKI, the inhibition of class I HDACs reduced kidney damage through regulating oxidative stress, apoptosis, and inflammation.

The role of class I HDACs in different AKI models is controversial (Figure 1). This may be due to model specific or differences in the HDACi structure. In addition, the time and dose of administration may also affect the function of class I HDACis in AKI. In aristolochic acid (AA)-induced AKI, since renal tubular repair is delayed in this model compared with IR-AKI, M4PTB treatment was performed on the fourth day after AA injury, which could increase proliferation and decrease G2/M arrest of regenerating renal tubular epithelial cells (Novitskaya et al., 2014). In the IRI model and cold ischemia renal transplant model, MS-275 was administered 16 h before modeling, and the treatment with MS-275 (3 mg/kg) could protect renal function in the early stage after injury and was associated with a substantial diminution of fibrosis long term after AKI (Levine et al., 2015). In our study (Tang et al., 2014), MS-275 (20 mg/kg) was given intraperitoneally immediately after folic acid injection or 2 h after glycerol injection and then administered daily, and we obtained different results. Measurement time may also affect the result. Hyndman et al. (2019) found that, following IRI, there was a significant increase in HDAC1 and HDAC4 by 72 h of reperfusion. Samples were taken at 72 h of reperfusion, and MS-275 treatment resulted in a significant increase in plasma creatinine and renal damage. In another study, samples were taken at 24 h of reperfusion, and MS-275 was proved to be renoprotective (Zhang et al., 2018).

## Class II Histone Deacetylases and Acute Kidney Injury

Class II HDACs include HDAC4, 5, 6, 7, 9, and 10. In our previous studies (Tang et al., 2018), we found that blocking HDAC6 with tubastatin A (TA) can reduce renal injury and improve renal function. Further studies showed that TA enhanced Akt phosphorylation and retained E-cadherin expression. Inhibition of HDAC6 also enhanced the expression of autophagy and pro-inflammatory cytokines. These data suggest that inhibition of HDAC6 has a protective effect on cisplatin-induced AKI. In a murine model of AKI induced by rhabdomyolysis, we also found that inhibition of HDAC6 activity prevented rhabdomyolysis-induced AKI (Shi et al., 2017). Inhibition of HDAC6 with TA resulted in a significant increase in AMWAP expression in proximal tubular cells, further reducing inflammation and apoptosis (Ranganathan et al., 2016). In another study on rhabdomyolysis-induced AKI (Feng et al., 2018), administration of compound 23BB, a highly selective HDAC6 inhibitor, improved kidney function and alleviated renal tubular injury. Mechanistic studies show that 23BB exerts potent renoprotective effects by inhibition of tubular cell apoptosis through a mechanism involved in the inactivation of ER stress.

After IRI, HDAC4 expression in the kidney is increased, and HDAC4 activation promotes the proliferation of renal tubular cells (Hyndman et al., 2019). Recently, we investigated the effect of selective class IIA HDAC inhibitor TMP195 on LPS-induced AKI in a murine model (Zhang et al., 2020). Treatment with TMP195 significantly reduced the kidney injury, which was consistent with decreased HDAC4 expression. TMP195 could also reduce the tubule damage and the apoptosis of renal tubular cells through reversing the increased expression of BAX/caspase-3 and decreased expression of Bcl-2. In addition, TMP195 increased the expression of BMP7 and reduced the upregulation of multiple pro-inflammatory cytokines/chemokines. Collectively, these data reveal that TMP195 was renoprotective in septic AKI.

Marumo et al. revealed that renal ischemia/reperfusion in mice induced a decrease in histone acetylation in renal tubular cells, which may be due to decreased histone acetyltransferase activity in cultured energy-depleted renal epithelial cells. HDAC5 was selectively down-regulated in parallel with the recovery of acetylated histones during recovery from transient energy depletion in epithelial cells. RNAi inhibition of HDAC5 significantly increased histone acetylation and BMP7 expression (Marumo et al., 2008). BMP7 is a renal protective protein that promotes the regeneration and repair of tubular epithelial cells after renal ischemia injury (Villanueva et al., 2006; Tomita et al., 2013). BMP-7 can also reverse TGF-β1–induced epithelial-mesenchymal transition by reinduction of E-cadherin or directly antagonizing the Smad signaling pathway (Zeisberg et al., 2003). In summary, this study suggests that downregulation of HDAC5 activates post-ischemic regeneration and alleviates renal injury by inducing BMP7 expression (Marumo et al., 2008). In addition, Hsing et al. found that decreased expression levels of HDAC2 and HDAC5 in septic AKI were associated with increased BMP-7 and acetyl histone H3 expressions (Hsing et al., 2012).

Trichostatin A (TSA), a pan-HDACi (class I/II), can prevent TGF-β1–induced EMT in cultured human renal proximal tubular epithelial cells through inducing several inhibitory factors of TGF-β1 signals, such as inhibitors of DNA binding/differentiation 2 and BMP-7, instead of altering the phosphorylation of Smad2 and Smad3 (Yoshikawa et al., 2007). In cisplatin-induced AKI models, TSA administration suppressed cisplatin-induced kidney inflammation and tubular epithelial cell apoptosis through upregulating AMWAP expression (Ranganathan et al., 2016). TSA can also protect the kidneys in cisplatin-induced AKI by enhancing autophagy (Liu et al., 2018). VPA (class I/II HDACi) can ameliorate kidney IR injury *via* reduced inflammatory cytokines, apoptosis/stress-related gene expression, and improved regeneration (Speir et al., 2015). VPA can induce the expression of BMP-7 and inhibit the apoptosis of epithelial cells to reduce the nephrotoxicity of cisplatin (Ma et al., 2017). Recent studies have shown that VPA can also attenuate hypertonic glycerol–induced rhabdomyolysis and acute kidney injury (Hareedy et al., 2021).

## Class III Histone Deacetylases and Acute Kidney Injury

Mammals have seven different silencing proteins (SIRT1–7); sirtuins are expressed in different subcellular compartments and modulate different cellular and biological functions to influence renal injury: SIRT3 and SIRT5 are mitochondrial sirtuins; SIRT1, SIRT6, and SIRT7 are nuclear sirtuins; and SIRT2 shuttles between the nucleus and the cytoplasm (Peasley et al., 2021).

In the kidney, SIRT1 is widely expressed in tubular cells and podocytes; it contributes to maintaining renal homeostasis, and its downregulation leads to chronic and acute kidney diseases (Morigi et al., 2018). In a variety of AKI animal models (including sepsis, I/R, cisplatin-induced AKI models), activation of SIRT1 can reduce renal injury. Inflammation is one of the main mechanisms responsible for the progression of AKI. SIRT1 activation can attenuate inflammation by interacting with high-mobility group box 1 (HMGB1) at the deacetylated lysine sites K28, K29, and K30 (Wei et al., 2019), and suppressing pro-inflammatory cytokine (TNF-α, IL-6) production (Chou et al., 2021), vascular cell adhesion molecule-1 (VCAM-1) and intercellular adhesion molecule-1 (ICAM-1) expressions, STAT3/ERK phosphorylation, and nuclear factor kappa-B (NF-κB) activation (Gao et al., 2014). SIRT1 overexpression can suppress the acetylation of NF-κB/p65 at lysine 310 in cisplatin-treated renal tubular cells (Kim et al., 2019). SIRT1 can also inhibit apoptosis by deacetylation of several apoptosis-related proteins, including p53 (Lys379), Smad7, forkhead box O3 (FOXO3), and FOXO4 (Kim et al., 2011; Fan et al., 2013; Kim et al., 2019). Autophagy (Atg) protects the kidney from acute I/R injury as well as cisplatin-induced nephrotoxicity (Kaushal, 2012). SIRT1 can induce autophagy by directly deacetylating autophagy-related 5, 7, 8 genes (Lee et al., 2008), and forming deacetylated Atg5, Atg7, and Atg8. SIRT1 activation can also induce autophagy by promoting the deacetylation of Beclin1 at K430 and K437 (Deng et al., 2021). Oxidative stress is closely associated with cisplatin-induced AKI; in cisplatin-induced AKI, kidney damage caused by cisplatin reduces the number and function of mitochondria and increases the production of ROS. SIRT1 overexpression in proximal tubules rescues cisplatin-induced AKI by maintaining peroxisome number and function and eliminating renal ROS levels, increasing purine degradation, and promoting ATP generation (Hasegawa et al., 2010; Kim et al., 2019). The second mechanism, SIRT1, promotes PGC-1 transcription and expedites recovery of mitochondrial protein expression and function by enhancing MB (Funk and Schnellmann, 2013). In a recent study, the female sex hormone is suggested to be renoprotective, and SIRT1 is necessary to regulate the estrogen receptor-α (ER-α) signaling pathway; inhibition of SIRT1 activity leads to inhibition of transcription of ER-α and its downstream genes (Darvishzadeh et al., 2021).

SIRT3 overexpression counterbalances the mitochondrial dysfunction through the reduction of mitochondrial fission factor (MFF)/dynamin-related protein (DRP1) expression balanced by upregulation of optic atrophy 1 (OPA1), preservation of Δψm, and inhibition of PTEN-induced putative kinase 1 (PINK1) (Morigi et al., 2015). Decreased SIRT3 expression is associated with increased severity of renal injury induced by I/R, and SIRT3 can be restored by the AMP-activated protein kinase (AMPK)/PGC-1 pathway to regulate mitochondrial homeostasis and reverse renal injury (Peerapanyasut et al., 2020). SIRT3 also acts as an antioxidant stress by deacetylating SOD2, P53, and FOXO3 (K271 and K290) to protect mitochondria against oxidative stress (Tseng et al., 2013; Ouyang et al., 2019). In sepsis-induced AKI models, SIRT3 can protect against mitochondrial damage in the kidney by reducing oxidative stress and inflammatory cytokines (Zhao et al., 2016). SIRT3 can also protect against sepsis-induced AKI by promoting autophagy via upregulating p-AMPK and downregulating phosphor-mammalian target of rapamycin (p-mTOR) (Zhao et al., 2018). SIRT3 deficiency can increase renal tubular apoptosis and inflammation and aggravate renal injury (Kim et al., 2018). Recent studies suggest that SIRT3 may regulate fatty acid oxidation (FAO) by deacetylating liver kinase B1 and activating AMP-activated protein kinase to reduce cisplatin-induced AKI in mice (Li et al., 2020).

SIRT6 expression was down-regulated in the kidney of IRI mice, and SIRT6 overexpression inhibited G2/M phase arrest and attenuated hypoxia-induced tubular epithelial cell damage (Gao et al., 2020). SIRT6 attenuates cisplatin-induced kidney injury by repressing the expression of ERK1/2 through deacetylating histone 3 at Lys9, thus inhibiting NF-κB and p53 signaling (Li et al., 2018). SIRT6 can also attenuate cisplatin-induced kidney injury by reducing oxidative stress and apoptosis via activating the SIRT6/Nrf-2 pathway (Li et al., 2018).

Compared with SIRT1, SIRT3, and SIRT6, depletion of SIRT2 and SIRT7 significantly improves cisplatin-induced AKI. SIRT7 regulates the inflammatory response through modulating NF-κB transcription activity and nuclear translocation (Miyasato et al., 2018). A decrease in SIRT2 increases the acetylation of mitogen-activated protein kinase phosphatase-1 (MKP-1) and thus suppresses the cisplatin-induced phosphorylation of p38 and C-Jun N-terminal kinase (JNK) in the kidney and tubular epithelial cells (Jung et al., 2020). The role of SIRT5 in cisplatin-induced AKI has been debated, and its exact role in AKI needs to be further clarified.

**FIGURE 2:**
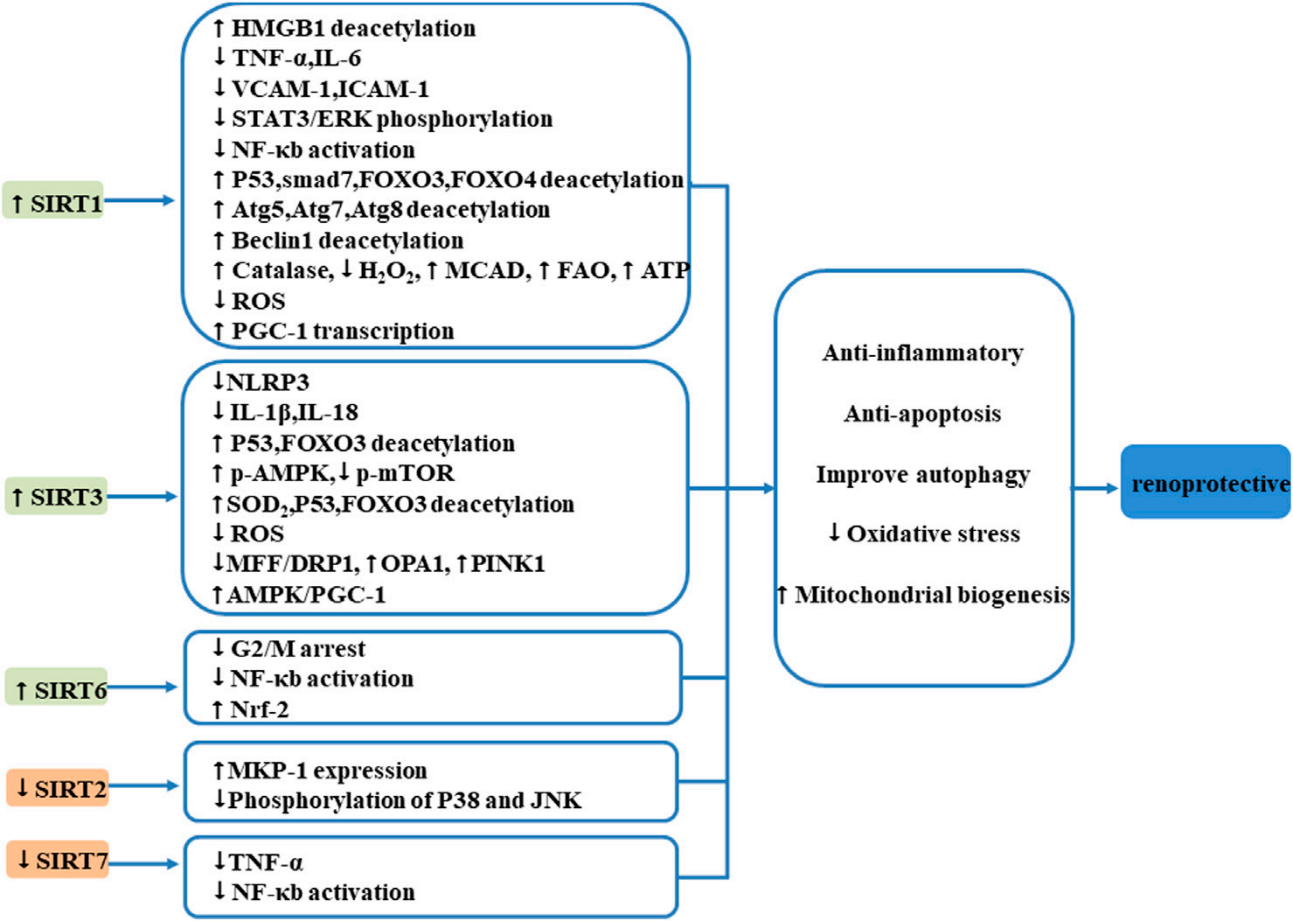
Mechanism of SIRTs influencing the kidney after AKI. Sirtuins play critical roles in cellular homeostasis, overexpression of SIRT1, SIRT3, and SIRT6, and suppression of SIRT7; SIRT2 is renoprotective. Sirtuins participate in acute kidney diseases through regulation of oxidative stress, apoptosis, inflammation, autophagy, and MB.

In general, sirtuins play critical roles in cellular homeostasis, overexpression of SIRT1, SIRT3, and SIRT6, and suppression of SIRT7 and SIRT2 is renoprotective, and numerous published studies have revealed that sirtuins participate in acute kidney diseases through regulation of oxidative stress, apoptosis, inflammation, autophagy, and MB (Figure 2).

## Class IV Histone Deacetylase and Acute Kidney Injury

HDAC11 is the only member of class IV. In the AKI-induced model, HDAC11 binds to the promoter region of PAI-1, which is then released by I/R insult. HDAC11 gene silencing increased PAI-1 expression, which can induce inflammation and functional loss (Kim et al., 2013). It is suggested that HDAC11 may be a new target for I/R damage.

## Conclusion and Perspectives

In this review, we summarized the roles and mechanisms of HDACs in AKI. HDACs are involved in renal injury by regulating inflammation, fibrosis, cell proliferation, apoptosis, autophagy, and oxidative stress. The main regulatory mechanisms include TGF/Smad signaling pathway, EGFR signaling pathway, pro-inflammatory cytokines release, NF-κB pathway, and G2/M arrest. Due to their specific location, SIRTs are also involved in renal injury by regulating mitochondrial function.

Previous studies have shown that there are sex-specific HDAC expression differences in brain regions (Gilbert et al., 2019), and sexually dimorphic expression of HDACs could be relevant to the higher prevalence of neuropsychiatric disorders and neurodegenerative diseases in females. In the kidney, HDAC9 mRNA and protein levels in the renal cortex of female rats were higher than those of male rats, while other HDAC levels did not differ by sex; HDAC9 is a novel inhibitor of augmentation of renal proximal tubular angiotensinogen (AGT) regulation, leading to low levels of AGT in the kidneys of women, protecting females from hypertension and related end-organ damage (Bourgeois et al., 2017). These studies all suggest that there are differences in HDAC expression in different genders, which may affect the disease incidence. Whether gender affects the role and mechanism of HDACs in AKI has not been reported yet. Therefore, we also included gender in Table 1, hoping that it could be helpful for subsequent studies.

A number of structurally diverse HDACis have been identified, and many are being evaluated in clinical trials, especially for cancer treatment (Ceccacci and Minucci, 2016). In 2003, Mishra et al. (2003) first showed that TSA treatment can significantly improve renal damage in lupus nephritis. Since then, various HDACis have been investigated for their antifibrotic and anti-inflammatory effects on renal diseases. HDACis have been shown to be effective in treating a variety of kidney diseases in animal models (Liu and Zhuang, 2015); however, due to their adverse reactions, such as hyponatremia, hypokalemia, edema, and changes in blood pressure (Hyndman et al., 2020), their application in clinical trials has been limited. This is probably because most of the research in this area is currently being done using pan-HDACis or class-specific HDACis. Broad-spectrum HDACi is more likely to cause nephrotoxicity, so specific HDAC inhibitors need to be developed to improve clinical efficacy and reduce toxicity. The typical HDACs consist of a cap, a linker, and a zinc-binding group. Modifying the cap and changing the linker or the zinc-binding group are common strategies for improving selectivity. In addition, attention should be paid to improve the drug-like properties of selective HDACis, such as drug delivery and *in vivo* bioavailability, as well as pharmacodynamics characteristics, to enhance their suitability for clinical applications. Combination therapy by combining HDAC inhibitors with other drugs may also benefit from this treatment. The body of HDACis still gets growing, with many new chemicals derived from natural products found to be potential HDACis. Although there have been no clinical trials of HDACis against AKI, we believe that HDAC-targeted regulation of protein acetylation has certain therapeutic potential in the treatment of AKI in the future.

## Glossary

AA: aristolochic acid
AGT: augmentation of renal proximal tubular angiotensinogen
AKI: acute kidney injury
AMPK: AMP-activated protein kinase
AMWAP: activated microglia/macrophage WAP domain protein
Atg: autophagy
ATP: adenosine triphosphate
BMP-7: bone morphogenetic protein-7
DRP1: dynamin-related protein 1
EGFR: epidermal growth factor receptor
ER: endoplasmic reticulum
ER-α: estrogen receptor-α
FAO: fatty acid oxidation
FOXO3: forkhead box O3
HATs: histone acetyltransferases
HDACi: histone deacetylase inhibitor
HDACs: histone deacetylases
HMGB1: high-mobility group box 1
ICAM-1: intercellular adhesion molecule-1
IRI: ischemia reperfusion injury
JNK: C-Jun N-terminal kinase
LPS: lipopolysaccharide
MB: mitochondrial biogenesis
MFF: mitochondrial fission factor
MKP-1: mitogen-activated protein kinase phosphatase-1
mTOR: mammalian target of rapamycin
NF-κB: nuclear factor kappa-B
OPA1: optic atrophy 1
PCNA: proliferative cell nuclear antigen
PINK1: PTEN-induced putative kinase 1
ROS: reactive oxidative species
RPTC: renal proximal tubular cell
STAT3: signal transduction and transcriptional activator 3
TA: tubastatin A
TSA: trichostatin A
VCAM-1: vascular cell adhesion molecule-1
